# Understanding Depression as It Occurs in the Context of Post-Traumatic Stress Disorder

**DOI:** 10.1155/2012/178261

**Published:** 2012-07-18

**Authors:** Rachel Yehuda, Eric Vermetten, Alexander McFarlane

**Affiliations:** ^1^Division of Traumatic Stress Studies, Department of Psychiatry, Mount Sinai School of Medicine, USA; ^2^The University of Queensland, Australia

The aim of the current special issue is to gain a better understanding of depression as it occurs in the context of posttraumatic stress disorder (PTSD). Depression and major depressive disorder (MDD) are often present in the context of PTSD, but it is not clear whether the high prevalence of depressive disorders in PTSD should be best understood as true comorbid conditions, with a separate underlying cause and pathophysiology or rather, whether it should best be considered an artifact of overlapping symptoms. Indeed, when individuals with PTSD also meet criteria for depression, this may signal the presence of a single depressive subtype of PTSD rather than two separate syndromes. Epidemiologic data support the idea that a depressive diathesis can be a risk factor for the development of PTSD following trauma exposure. However, the question of childhood trauma as an even earlier antecedent of depression suggests that the depression that contributes to PTSD risk may relate to earlier forms of trauma exposure rather than genetic diatheses per se. Prospective studies assessing the development of major depressive disorder (MDD) show that, although the prevalence of PTSD is higher shortly after exposure, later on approximately the same prevalences of PTSD and MDD are seen after exposure to traumatic events. Severe depressive symptoms in the early aftermath of trauma exposure can be considered more relevant in predicting an unfavorable course of mental health than severity of PTSD symptoms, underlining the relevance of broad mental health assessment after trauma exposure. Whether depression in PTSD represents a phenomenological expression of the same underlying pathophysiology becomes interesting to consider in the context of the rather distinct biological alterations that have been observed in PTSD and major depressive disorder, particularly in the context of neuroendocrine, neurochemical, and brain metabolic factors. Beside clinical characteristics, biological markers may help to further improve identification of biologically distinct endophenotypes and, ultimately, to devise more specific treatment strategies. Although antidepressants are among the only FDA-approved pharmacological treatments for PTSD, these medications have only limited utility in PTSD, and it is not clear whether the efficacy of these agents results from the treatment of comorbid depression or PTSD per se.

In this special issue we have pulled together a collection of papers that help broaden the scope and understanding of depression in the context of adversity. Papers are also included that present remarkably innovative treatment approaches for trauma-related symptoms.

The first paper by J. D. Flory et al. asks the interesting question of what are the effects on adults of familial depression in the context of childhood abuse. This is an extremely significant question because both a history of MDD and childhood abuse have been independently identified as risk factors for adult PTSD and MDD. The causal relationship between parental depression and childhood abuse has not been firmly made in the literature, but the results of this observational study suggest that, when persons are exposed to both depression in their first-degree relatives and childhood abuse, the risk for adult PTSD is sixfold higher relative to the absence of both of these factors. The rate of depression in the presence of both factors was also greater with these combined factors. These data suggest that the risk factors for PTSD and possibly depression are not cumulative but may be synergistic. Possibly the data reflect the permissive effect of depression on the environment; for example, a depressed parent may be unable to monitor the environment of the child sufficiently to protect against abuse. Alternatively, advances in molecular biology might further test this hypothesis by determining whether synergist effects are mediated by underlying biological mechanism.

The idea of identifying specific risk factors for PTSD versus posttraumatic depression is also examined in the paper by G. Buodo et al., but here a different approach is taken. This group evaluated symptoms in workers who were directly exposed to traumatic occupational accidents. The paper demonstrates that nearly half the sample developed both PTSD and major depressive disorder, 30% did not develop symptoms, and 20% developed symptoms only of PTSD. The findings raise the question of whether posttraumatic depression is an amplifier of posttraumatic stress disorder or represents the expression of a pre-existing depression diathesis. Though this question does not get resolved by these cross-sectional data, the authors attempt to relate different symptom patterns with different posttraumatic cognitive profiles and suggest that specific posttraumatic cognitions underlying PTSD and depression may demonstrate a distinct etiology of these two comorbid conditions.

Together, the papers by Flory et al. and Buodo et al. raise the question of whether depression occurring in the context of PTSD or trauma requires a specifically focused mental health intervention. The paper by McFarlane and Williams discusses the type of mental health services that are required following disasters and how these need to take account of the spectrum of distress that occurs following such events. In particular, disasters are a specific example of how traumatic events impact on individuals and populations. In the past, the literature has tended to ignore the background prevalence of posttraumatic stress disorder, depression, and other psychiatric disorders. In essence, this paper discusses how service delivery and planning needs to take account of the fact that PTSD is only one outcome of clinical relevance following traumatic exposures. Furthermore, a variety of environmental factors impact upon the progression and delayed emergence of PTSD and depression in the aftermath of such events that need to be taken into account in the development of service delivery models. The prevalence of traumatic events and the significant psychiatric morbidity that they generate need to be addressed in the planning of mental health services because of the substantial associated burden of disease that often goes untreated. 

Two other papers in this special issue also focus on treatment of trauma survivors but take very different perspectives from that detailed by A. McFarlane and R. Williams and, in fact, different from each other. J. A. Golier et al. report promising preliminary findings from a pilot study of mifepristone in combat-related PTSD. Mifepristone is a synthetic steroid compound that primarily functions as a progesterone receptor antagonist and has been primarily used, up until now, as an abortifacient. The compound is also known as RU-486. However, as Golier and colleagues point out, mifepristone also has properties as a selective antagonist of glucocorticoid receptors, and therefore, this drug induces increases in cortisol and ACTH levels by blocking the negative feedback inhibition of the hypothalamic-pituitary-adrenal axis peripherally and in the brain. Although the mechanisms of action by which mifepristone might exert its therapeutic actions are not completely understood, it is fascinating, in the context of the previous papers regarding depression and PTSD comorbidity, that this drug was first considered as a treatment for depression. Indeed, the blockade of GR receptors could arguably decrease the effect of peripheral cortisol, which is elevated in depression but thought to be lower in PTSD. However, once in the brain, mifepristone retards the rate at which brain cortisol is expelled at the blood-brain barrier effectively increasing cortisol levels in the brain. After taking the drug for one month, which constituted the treatment endpoint, the effects of mifepristone were clinically significant and were even greater at the four-week followup. Since neuroendocrine data were obtained, the authors could determine that mifepristone increased cortisol and ACTH levels and decreased the number of peripheral glucocorticoid receptors, raising the possibility of enhanced glucocorticoid signaling in cell nuclei. The findings from this approach are important to understanding how altering the neuroendocrine status of hormones associated with both PTSD and depression can lead to therapeutic gain.

Finally, the paper by S. Telles et al. discusses a completely different strategy for managing mental health symptoms following trauma—the use of yoga. The authors argue that yoga exerts its therapeutic benefits largely through altering physiological states. Indeed, yoga involves physical manipulation of the body through postures, voluntary-regulated breathing, meditation, conscious sensory withdrawal, and changes in thinking. There have only been eleven studies in which yoga with medication was used as a trauma-related intervention. The authors grapple with the fact that this is an emerging field and the studies that have been documented in the literature to date are preliminary and sometimes lacking in gold-standard methodologies that typify other therapeutic intervention studies. Yet based on their review, there appears to be sufficient anecdotal evidence for yoga's benefits and a sufficient rationale for how yoga practice may alter the important neurochemical systems that are altered in response to traumatic stress. This is clearly an area worthy of further research.

As the aim of this special issue was to gain a better understanding of depression as it occurs in the context of trauma exposure and PTSD, the research in this special issue covers support for the notion that major depressive disorder is often comorbid with PTSD and that there is also supportive evidence both for the fact that depression in PTSD can be understood as a true concomitant condition as well as having a separate underlying cause and pathophysiology. As posttraumatic symptoms are often neglected in depression focused research and clinical care, future effort should address the recognition and treatment of posttraumatic symptoms in depression. This opens avenues for new treatment perspectives of which some are outlined in this special issue as well.

*Rachel Yehuda* *Rachel Yehuda* *Eric Vermetten* *Eric Vermetten* *Alexander McFarlane* *Alexander McFarlane*

